# Supplementary figures and images for: MAPE-ViT: multimodal scene understanding with novel wavelet-augmented Vision Transformer
Source: PeerJ Comput Sci. 2025 May 23;11:e2796. doi: 10.7717/peerj-cs.2796 (PMC12190338; doi:10.7717/peerj-cs.2796)

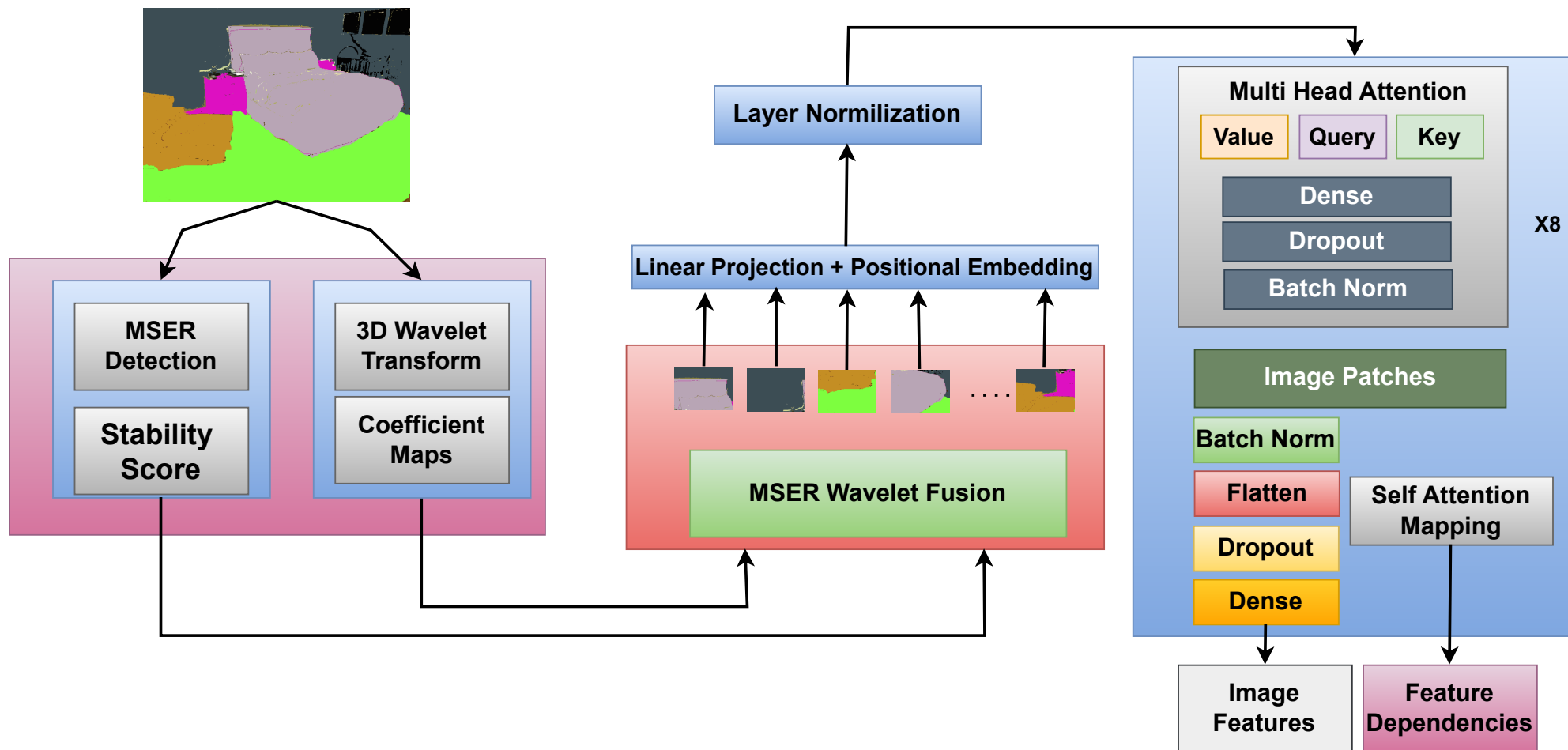

Supplement: Supplemental Information 2 [file peerj-cs-11-2796-s002.pdf]

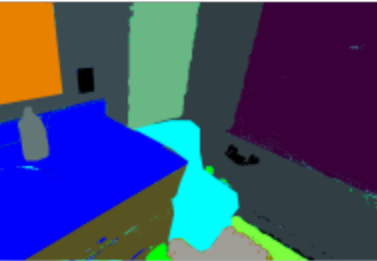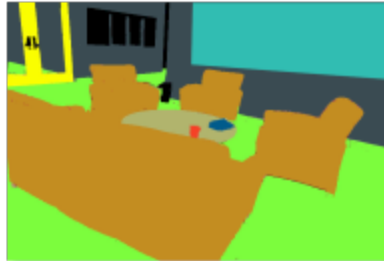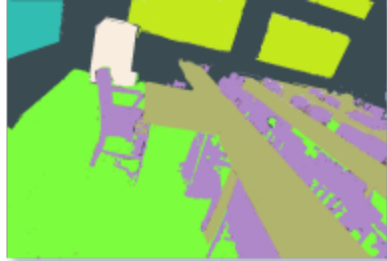

Basin

Chair

Wall

Towel

Cup

Mirror

Floor

Board

Sofa

Dice

Table

Book

Door

Window

Commode

Supplement: Supplemental Information 3 [file peerj-cs-11-2796-s003.pdf]

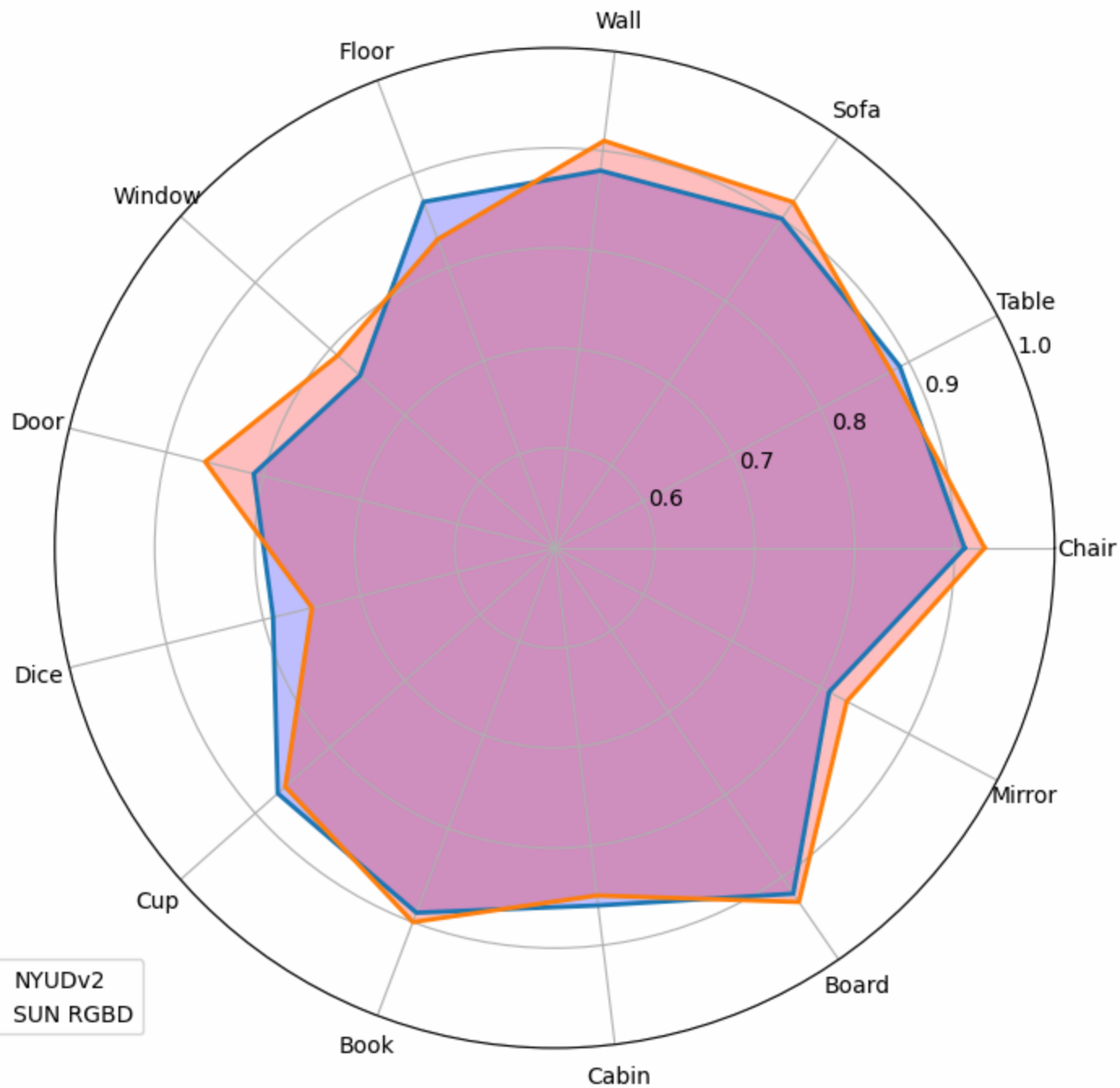

Supplement: Supplemental Information 4 [file peerj-cs-11-2796-s004.pdf]
