# Supplementary material for: MAPE-ViT: multimodal scene understanding with novel wavelet-augmented Vision Transformer
Source: PeerJ Comput Sci. 2025 May 23;11:e2796. doi: 10.7717/peerj-cs.2796 (PMC12190338; doi:10.7717/peerj-cs.2796)
Supplement: Supplemental Information 5 [file peerj-cs-11-2796-s005.docx]

**Computing infrastructure**

The experiments were conducted on a PC equipped with an x64-based Windows 10 operating system, an Intel Core i3-4010U 1.70.GHz CPU, 4GB RAM. The system’s performance was evaluated using two benchmark datasets: SUN RGB-D and NYU v2.
