# Supplementary material for: MAPE-ViT: multimodal scene understanding with novel wavelet-augmented Vision Transformer
Source: PeerJ Comput Sci. 2025 May 23;11:e2796. doi: 10.7717/peerj-cs.2796 (PMC12190338; doi:10.7717/peerj-cs.2796)
Supplement: Supplemental Information 6 [file peerj-cs-11-2796-s006.docx]

**MAPE-ViT: Multimodal Scene Understanding with Wavelet-Augmented Vision Transformer**

**Overview**

**MAPE-ViT** is a model designed for multimodal scene understanding by combining RGB and depth data. The model processes images through several stages, including preprocessing, fusion, segmentation, feature extraction, multi-object classification, and scene classification. By leveraging the strengths of the Vision Transformer, Extreme Learning Machine, and Conditional Random Fields, this model aims to improve accuracy in both object segmentation and scene classification.

**Table of Contents**

1. [Project Setup](#project-setup)
2. [Data Preparation](#data-preparation)
3. [Code Explanation](#code-explanation)
4. [Implementation Steps](#implementation-steps)
5. [Visualization](#visualization)
6. [Additional Notes](#additional-notes)
7. [License](#license)

**Project Setup**

**Prerequisites**

- **Python 3.x**
- **Libraries**: Install the required libraries by running:

*install torch torchvision opencv-python numpy scipy matplotlib*

**System Requirements**

This code has been tested on Windows 10 with the following configuration:

- **CPU**: Intel Core i3-4010U
- **RAM**: 4GB

For optimal performance, consider using a machine with higher specifications.

**Data Preparation**

Download the SUN RGB-D and NYU v2 datasets and place them in a directory named datasets within the project root. The paths to RGB and depth images should match the requirements in the code. If needed, modify the *load_and_preprocess* function to adjust the image paths.

**Code Explanation**

The model consists of several key components, each implemented within the provided code:

1. **Preprocessing**: The *load_and_preprocess* function loads and resizes the RGB and depth images, normalizes them, and converts them to tensors for processing.
2. **Fusion Model**: *FuseNe*t combines RGB and depth channels by passing them through separate convolutional layers and then fusing them into a single output. This fusion provides a more informative input for segmentation.
3. **Multi-Dimensional Gradient-Aware Segmentation**:
   - The *gradient_aware_segmentation* function segments the fused image by leveraging spatial, color, and gradient information.
   - Functions such as *compute_spatial_distance, compute_color_distance*, and *compute_gradient_distance* are used to calculate distances between pixels based on these features.
4. **Feature Extraction with MAPE-ViT**:
   - **MSER Detection**: The *mser_detection* function identifies regions using MSER, which are then processed using wavelet transforms (*wavelet_transform*).
   - **Adaptive Patch Embedding**: The *adaptive_patch_embedding* function embeds patches with features from both MSER and wavelet transforms, preparing them for the Vision Transformer.
   - The *SegmentTransformer* is a transformer-based model that learns segment-level features from these patches.
5. **Multi-Object Classification with ELM**:
   - The Extreme Learning Machine classifier receives features from the Vision Transformer for classifying objects within each scene.
6. **Scene Classification with Conditional Random Fields**:
   - *CRFSceneClassifier* integrates information from ELM, Vision Transformer features, and depth data to classify the overall scene context using a CRF.

**Implementation Steps**

**Step 1: Preprocess the Images**

Run the preprocessing function to prepare the RGB and depth images:

*rgb_tensor, depth_tensor = load_and_preprocess('path_to_rgb_image', 'path_to_depth_image')*

**Step 2: Fuse RGB and Depth Data**

Fuse the preprocessed images to enhance their information content:

*fused_image = FuseNet()(rgb_tensor, depth_tensor)*

**Step 3: Segment the Fused Image**

Use the gradient-aware segmentation method to partition the fused image:

*segmented_image = gradient_aware_segmentation(fused_image.numpy())*

**Step 4: Extract Features with MAPE-ViT**

- Apply MSER and wavelet transforms for feature extraction:

*regions = mser_detection(segmented_image)*

*wavelet_coeffs = wavelet_transform(regions[0])*

- Perform adaptive patch embedding and pass through the Vision Transformer:

*patch_embeddings = adaptive_patch_embedding([regions[0]], [wavelet_coeffs])*

*segment_features = SegmentTransformer(d_model=patch_embeddings.shape[1], num_layers=2)(patch_embeddings)*

**Step 5: Classify Objects with ELM**

- Use the extracted segment features to classify objects:

*`elm_probs` are obtained from an ELM model here*

**Step 6: Classify the Scene with CRF**

- Integrate the multi-object classification results and depth data for scene classification:

*scene_labels = classify_scene(elm_probs, segment_features, depth_grad_stats, num_classes)*

**Step 7: Save and Load Model Parameters**

To save the trained model:

*torch.save(model.state_dict(), 'mape_vit_model.pth')*

To load the model for further evaluation:

*model = CRFSceneClassifier(num_classes=5, input_dim=feature_dim)*

*model.load_state_dict(torch.load('mape_vit_model.pth'))*

*model.eval()*

**Visualization**

To visualize RGB, depth, and fused images, use:

*visualize_results(rgb_tensor, depth_tensor, fused_image)*

For initial segmentation and MSER detection:

*initial_segmentation(rgb_image)*

*mser_detection(segmented_image)*
